# Perioperative Nutritional Support: A Review of Current Literature

**DOI:** 10.3390/nu14081601

**Published:** 2022-04-12

**Authors:** Antonio Jesús Martínez-Ortega, Ana Piñar-Gutiérrez, Pilar Serrano-Aguayo, Irene González-Navarro, Pablo Jesús Remón-Ruíz, José Luís Pereira-Cunill, Pedro Pablo García-Luna

**Affiliations:** Unidad de Gestión Clínica de Endocrinología y Nutrición, Instituto de Biomedicina de Sevilla (IBiS), Hospital Universitario Virgen del Rocío/CSIC/Universidad de Sevilla, 41013 Seville, Spain; ajesus.martinez.sspa@juntadeandalucia.es (A.J.M.-O.); anapinarg@gmail.com (A.P.-G.); mariap.serrano.sspa@juntadeandalucia.es (P.S.-A.); irenegonzalez1@gmail.com (I.G.-N.); pjremonruiz@gmail.com (P.J.R.-R.); garcialunapp@yahoo.es (P.P.G.-L.)

**Keywords:** perioperative nutritional support, Enhanced Recovery After Surgery, enteral nutrition, immunonutrition, oncological surgery

## Abstract

Since the beginning of the practice of surgery, the reduction of postoperative complications and early recovery have been two of the fundamental pillars that have driven the improvement of surgical techniques and perioperative management. Despite great advances in these fields, the rationalization of antibiotic prophylaxis, and other important innovations, postoperative recovery (especially in elderly patients, oncological pathology or digestive or head and neck surgery) is tortuous. This can be explained by several reasons, among which, malnutrition has a major role. Perioperative nutritional support, included within the ERAS (Enhanced Recovery After Surgery) protocol, has proven to be a main element and a critical step to achieve better surgical results. Starting with the preoperative nutritional assessment and treatment in elective surgery, we can improve nutritional status using oral supplements and immunomodulatory formulas. If we add early nutritional support in the postoperative scenario, we are able to significantly reduce infectious complications, need for intensive care unit (ICU) and hospital stay, costs, and mortality. Throughout this review, we will review the latest developments and the available literature.

## 1. Introduction

Since the beginning of the practice of surgery, the reduction of postoperative complications and early recovery have been two of the fundamental pillars that have driven the improvement of surgical techniques and perioperative management. Despite great advances in these fields, the rationalization of antibiotic prophylaxis, and other important innovations, postoperative recovery (especially in elderly patients, oncological pathology or digestive or head and neck surgery) is tortuous. This can be explained by several reasons, among which, we highlight malnutrition, both preoperative and as a result of the surgical act itself, which substantially increases hospital stay and costs [1,2,3,4]. In 2005, the ERAS (Enhanced Recovery After Surgery) protocol was established for the first time for patients undergoing colonic surgery [5], with 22 recommendations, including the improvement of perioperative nutrition. The results since its implementation have been quite favorable, with significant reductions in postoperative complications and hospital stay [6,7]. In this sense, the improvement of the nutritional management of surgical patients has played a key role, being one of the main contributing factors to the success of the protocol [8,9]. Throughout this review, we will take a tour of the main clinical practice guidelines and provide an update on perioperative nutritional support in the pathologies with the greatest evidence.

## 2. Materials and Methods

A review of recent literature, published from January 2017 to February 2022, was performed. Three computerized electronic databases (PubMed, Web of Science, and Scopus) were searched using the following key search words: (“perioperative pathophysiology” OR “perioperative” OR “malnutrition” OR “head and neck cancer” OR “gastrointestinal cancer” OR “gastrointestinal surgery” OR “esophageal cancer” OR “bariatric surgery” OR “oncological nutrition” OR “oncological surgery” OR “colorectal cancer” OR “pancreatoduodenectomy” OR “spine surgery” OR “hip fracture” OR “bladder surgery”) AND (“Nutritional support” OR “immunonutrition” OR “Immune-Enhancing Enteral Therapy” OR “Enhanced Recovery After Surgery” OR “ERAS”) AND (“adults”). All possible articles were merged into a single file, and duplicate records were removed after they were checked manually.

## 3. Importance of Perioperative Nutrition and Physiopathology

Nutritional status is probably one of the most thoroughly studied and well-known determinants of surgical outcomes. Between 40 and 50% of patients undergoing surgery have some degree of malnutrition [10]. Preoperative malnutrition is associated with a higher rate of infections, worse evolution and healing of the surgical wound, development of pressure ulcers and prolonged hospital stay, both in the intensive care unit and in the conventional hospitalization plant [11,12,13,14,15,16,17,18,19,20]. Malnutrition is exacerbated by weight loss during hospitalization, which occurs in up to two-thirds of patients [21]. There are several mechanisms that justify this situation:

### 3.1. Mechanisms Related to the Disease That Indicates the Need for Surgical Intervention

In surgical patients, often of advanced age, there is a situation of chronic low-grade inflammation, sustained mainly by interleukin-6 (IL-6), interleukin-1 (IL-1), tumor necrosis factor alpha (TNF-α), interleukin-2 (IL-2) and interferon alpha (IFN-α), in addition to the nuclear factor kappa beta (NFκβ), target of rapamycin (TOR), transforming growth factor beta (TGF-β), Janus kinase/activator of transcription (JAK/STAT) and RAS pathway, among others [22,23,24,25]. These cytokines are expressed at above normal values, and their activation produces deleterious effects such as chronic inflammation, release of hepatic acute phase reactants, insulin resistance, increased catabolism with the appearance of sarcopenia (with the consequent decrease in muscle strength), and osteoclastic activity. Likewise, the intestinal microbiota seems to have a very important role in this process, together with nutritional status, in such a way that both malnutrition and overnutrition favor the pro-inflammatory environment. An oxidative environment also occurs in this context, which could affect the stability of DNA and its repair mechanisms. To counteract this inflammatory state, the pathway of the anti-inflammatory cytokines interleukin-4 (IL-4), interleukin-10 (IL-10) and interleukin-13 (IL-13) is activated inducing the stress response of the hypothalamic-pituitary-adrenal axis, which in turn induces an increase in cortisol synthesis that will cause secondarily, and as unwanted side effects, bone resorption, lipolysis, protein catabolism, gluconeogenesis and immune dysfunction, depending on the system on which it acts, ultimately producing frailty and sarcopenia. The coexistence of inflammatory and anti-inflammatory phenomena has a negative effect on metabolism, bone density, muscle mass and strength, exercise tolerance, vascular system, cognition and affect, ultimately helping to trigger the fragility phenotype, which frequently coexists with other geriatric syndromes [22,26,27,28,29]. All these mechanisms significantly increase the surgical risk and worsens the results [22,30].

On the other hand, we must not forget other factors potentially associated with worse preoperative nutritional status, such as intestinal obstruction (both by extrinsic and intrinsic compression), psychological suffering of the patient or prolonged hospitalization with fasting by diagnostic tests, among others [15,16,21,31,32].

### 3.2. Mechanisms Related to the Surgical Act

Surgery is, in itself, an aggressive procedure, which sets in motion inflammatory mechanisms similar to those described above, with secretion of the same pro-inflammatory cytokines and activation of neurohumoral mechanisms analogous to those that occur in malnutrition related to the disease, although on a larger scale and in a more intense way [33]. On the other hand, those interventions that affect the digestive tract (resections, anastomosis, etc.) usually impose important dietary restrictions initially on patients, which, together with prolonged bedding, increases sarcopenia and malnutrition [6,14,15,34,35,36,37,38].

## 4. Available Guidelines

As we have pointed out, perioperative nutrition is one of the fundamental principles behind the ERAS protocol. There are several clinical practice guides that address the subject, among which the European Society for Parenteral and Enteral Nutrition (ESPEN) and the ERAS Society stand out. The American Society for Parenteral and Enteral Nutrition (ASPEN) is currently preparing its own recommendations in this regard, which are expected to be available shortly.

### 4.1. ESPEN Guidelines

The latest ESPEN recommendations on perioperative nutritional management, published in 2021 [39], are based on the 2017 guidelines [40]. These are the same recommendations (specifically 37), although the presentation has been restructured to make it more accessible. It follows a methodology based on the Scottish Intercollegiate Guidelines Network (SIGN) system, giving a degree of recommendation (A/B/0/Good Practice Points (GPP)) based on the available evidence. A brief summary is available in Table 1.

### 4.2. ERAS Guidelines

The first reference to the ERAS protocol, as we have already mentioned, dates from 2005, in the form of a consensus document with 22 recommendations for patients undergoing colonic surgery [5]. Since then, numerous guidelines have emerged not only limited to this type of surgery, but also for esophageal–gastric, bariatric, pancreatic, gynecological, and traumatological surgery, among others, prepared by the different working groups of the ERAS society. At the present time, there are 24 guides available on the website of the ERAS society [41]. We will not make a detailed description of each of them, as it is not the objective of this review, but we will address some of them in certain sections. In summary, the ERAS protocol (also called pathway) is an evidence-based multimodal approach, the results of an integrated consensus on perioperative care that reorganizes care around surgery. The objective is to combine multiple evidence-based interventions, which in isolation can have a modest or small impact, within a structured program that allows synergies between them, in order to obtain an optimal result. Among these interventions, nutritional support has a leading role, along with prehabilitation [42].

## 5. Perioperative Nutritional Support in Oncological Pathology

One of the pathologies in which the role of perioperative nutritional support has been most studied is cancer. Due to the increased catabolism that occurs in this group of diseases, cancer patients have a high prevalence of malnutrition, which has been shown to have a great impact on their survival [43]. This is especially relevant when facing the different treatments they must undergo, including surgery [44]. Severe malnutrition is an independent risk factor for increased postoperative morbidity and mortality as well as longer length of hospital stay and higher cost in cancer patients [45]. Finally, it should be noted that the perioperative period has been shown to be critical in determining the risk of post-surgery metastasis, although there are no studies to date that evaluate the role of nutritional support in this regard [46].

When evaluating perioperative support in cancer patients, we must first consider which patients can benefit from it. In this regard, there are studies that support nutritional support only in patients with moderate or severe malnutrition or those at risk of not receiving adequate oral nutrition for at least 7–14 days after surgery [47]. This is because studies of malnourished patients have shown that nutritional support leads to a reduction in surgical complications, including infections [48,49,50]; whereas in studies in which all cancer patients were treated regardless of nutritional status and risk, well-nourished patients treated with parenteral nutrition had an increased risk of infections while malnourished patients had an overall benefit [51]. Studies comparing patients with and without perioperative nutritional support including all cancer patients also showed no benefit [52].

To elucidate who should be treated, it is important for hospitals to include nutritional screening in cancer patients who are going to undergo surgery. In this regard, studies have shown that there is a high percentage of surgeons who believe that there are data to support the optimization of preoperative nutritional status to reduce complications. However, in the US, only 1 in 5 hospitals perform nutritional screening of patients undergoing oncologic/gastrointestinal surgery and only 20% of these patients receive some type of nutritional supplementation in the pre- or postoperative period [36]. Similar results were obtained in 2021 in a UK study of anesthesiologists: although 91.7% of respondents agreed that perioperative nutrition improved quality of life after surgery, 49.6% acknowledged that they did not feel prepared to identify and manage patients in need of it [53].

Regarding the type of nutritional support to be used, the ESPEN recommends that oncology patients should also follow the ERAS guidelines [54], prioritizing the enteral nutrition. Enteral nutrition has been associated with improvements in nitrogen balance and weight gain in cancer patients [55]. Although parenteral nutrition (PN) has also demonstrated these benefits, weight gain with this type of support is due to an increase in fat [56]. On the other hand, there are studies in which PN has been associated with a higher incidence of infections and surgical complications and increased cost in oncology patients [57,58,59,60]. However, although no benefit has been demonstrated over enteral nutrition, there are studies that show a benefit over the use of perioperative fluid therapy in patients in whom the enteral route is not available [57,61]. On the other hand, there is controversy about the role of PN in increasing tumor proliferation, demonstrated in murine models [62], but with discordant results and with little clinical relevance in humans [63,64].

The role of immunonutrition in surgical oncology patients is also relevant. In 2009, the ASPEN guideline for nutritional support during anti-cancer treatments in adults [51] recommended the use of immunonutrients (specifically formulas with omega-3 acids, arginine and RNA) in cancer patients undergoing surgical treatment. The US Summit on Immune-Enhancing Enteral Therapy recommended the use of immunonutrient supplements in patients with malnourished gastrointestinal or head and neck cancer undergoing major surgery 5–7 days prior to surgery [65]. The ESPEN guidelines on nutrition in cancer patients and other authors recommend its use even in patients undergoing major gastrointestinal surgery regardless of their baseline nutritional status [54,66]. There are several studies supporting the use of these formulas in malnourished patients to reduce the length of hospital stay and the incidence of infectious complications and anastomotic leaks [48,67,68,69]. In terms of protein intake, whey protein and casein were shown in a study to be the best quality proteins for stimulating anabolism and protein synthesis in cancer patients [70].

Regarding the timing of nutrition initiation, the ASPEN guideline for nutritional support during anti-cancer treatments in adults [51] recommends that this be done in the 7–14 days prior to surgery, but always taking into account the risk of the nutritional support itself as well as the possible delays of the surgery.

### 5.1. Gastrointestinal Cancer

The evidence of perioperative nutritional support in cancer patients has been especially studied in gastrointestinal tumors, and the recommendations of the current guidelines can also be applied to non-oncological gastrointestinal surgery. A total of 65% of patients who undergo surgery for these tumors meet the criteria for malnutrition due to both tumor cachexia and decreased caloric intake [71].

In patients with gastric cancer, several perioperative nutritional parameters have been described as independent prognostic factors [72]. In a study of 800 patients with gastric cancer who underwent gastrectomy and were at high risk of malnutrition, the group with nutritional support for at least 10 days had fewer infections than the group with poor or no support [73]. In addition, early oral nutrition decreases length of hospital stay when compared to its delayed onset, with no increase in complications compared to those receiving parenteral nutrition [74]. Beyond surgical complications, the loss of weight and muscle mass in this period affects the toxicity of adjuvant treatment, which has implications for the continuity of these therapies, disease recurrence and survival [75]. Regarding the use of Oral Nutritional Supplements (ONS) in the perioperative period, their benefit may be diminished by the low compliance in this group of patients after the reduction of the size of their stomach [75].

The use of immunonutrition formulas in patients with gastric cancer who are going to undergo gastrectomy is controversial. Therefore, in 2016, Ma et al. [76] performed a systematic evaluation with 9 randomized clinical trials, concluding that omega-3 (n-3) polyunsaturated fatty acids (PUFA)-rich formulas significantly reduced postoperative infectious complications as well as length of hospital stay and duration of systemic inflammatory response syndrome, especially in malnourished patients.

In esophageal tumors, there are also studies showing that weight loss and/or skeletal and muscle mass loss are associated with worse treatment outcomes and increased recurrence at 5 years [77,78]. On the other hand, artificial nutrition has been associated with fewer post-surgical complications after esophagectomy [79]. As with gastrectomies, early oral nutrition is recommended, as there are safety data available [80]. Few studies have evaluated the role of eicosapentaenoic acid (EPA)-based immunonutrient formulations in preventing this loss, with discordant results [75]. Another issue under discussion is the role of vitamin D in patients with esophageal cancer undergoing esophagectomy, since two studies have shown that vitamin D supplementation or adequate vitamin D levels may be associated with a reduction in markers of alveolar edema and post-surgical lung injury [81,82].

In colorectal cancer (CRC), nutritional treatment on the first postoperative day was an independent predictor of survival at 5 years after surgery in the study by Gustafsson et al. [83]. There is also a growing interest in the role of immunonutrition and prebiotics, since alterations in the microbiota have been shown to be a risk factor for this type of tumor [84]. In 2011, a phase III clinical trial demonstrated that preoperative administration of prebiotics promoted recovery of intestinal function and improved both immune system function and nutritional status [85]. In 2018, another study demonstrated that the use of prebiotics improved both pre- and postoperative immunological parameters even if it did not alter the species of bacteria present in the microbiota [86]. In the same year, a meta-analysis evaluating immunonutrition in patients with CRC undergoing surgery was also published [87]. It showed that applying this type of formula via enteral nutrition reduced length of hospital stay and infectious complications, while parenteral nutrition reduced length of hospital stay and improved immunological parameters.

Patients with pancreatic cancer present with a high prevalence of malnutrition, measured both by weight loss and screening tools [88,89,90]. Surgery for this type of cancer is inherently at high risk of complications [91] and it has been classified as the surgery with the highest risk of 30-days readmission [92]. One of the most important risk factors for complications after pancreatic surgery is precisely malnutrition, which has been associated with a higher number of pancreatic fistulas [90,93], abdominal abscesses [88], hospital readmission [94], in-hospital mortality and 90-day mortality [88]. In fact, early identification of malnutrition and its treatment during admission has shown to reduce morbidity, length of stay and costs in these patients [90,95].

Several studies have been performed regarding perioperative nutritional support in patients undergoing pancreatoduodenectomy (PD). With respect to parenteral nutrition, the results obtained showed an increase in infectious complications, morbidity, length of stay and mortality when compared with intensive fluid therapy [96], standard enteral nutrition [97,98], enteral nutrition with immunonutrients [95,99], and oral diet [99]. On the other hand, enteral formulas with immunonutrients have also been compared with other enteral nutrition standard formulas, showing a decrease in postoperative infections and length of hospital stay [99,100,101]. In 2007, Bozzetti et al. [102] conducted a study in which postoperative complications were reduced in cancer patients undergoing PD with different degrees of malnutrition when fluid therapy was switched by parenteral nutrition, parenteral nutrition by standard enteral nutrition and the latter by enteral nutrition formulas with immunonutrients. Based on the results of these and other similar studies, ESPEN already in 2006 recommended the use of perioperative enteral nutrition (preferably with formulas containing immunonutrients) in patients undergoing PD (grade A) [103]. However, the ERAS guidelines for patients undergoing PD, updated in 2019 [104], recommend preoperative nutritional support in patients with severe weight loss, but do not recommend the use of immunonutrition formulas, with a high level of evidence and a strong grade of recommendation. This statement was based on the scarce data in patients undergoing PD and the heterogeneity of methodology and results in studies performed at present.

In 2016, Xiong et al. [105] performed a meta-analysis showing a decrease in morbidity and delayed gastric emptying in patients who followed ERAS protocols after undergoing PD, although this had not been demonstrated in a 2013 meta-analysis performed by Coolsen et al. [106]. In 2018, Ji et al. [107] conducted a new meta-analysis including 20 case-control studies with a total of 3694 patients, concluding that the implementation of ERAS protocols was safe and effective and could reduce postoperative complications. However, clinical practice studies show that the protocol established by the ERAS guidelines regarding early initiation of oral diet is not frequently followed, since its initiation is usually delayed and the progression of oral tolerance is usually slow, and the caloric requirements of the patients are not reached in the first days [108]. In 2014, Braga et al. [109] conducted a study in patients who underwent PD, comparing those who started liquids on 1st post operative day (POD) and solids on 2nd POD post-surgery vs. patients who started them on 3rd POD and 4th POD, respectively. The results showed that low compliance with ERAS targets was associated with a higher rate of complications and greater severity of complications. Therefore, authors such as Bozzetti [108] recommend the use of enteral nutrition by nasojejunal tube or jejunostomy (depending on the patient’s preferences) until the caloric and proteic requirements with oral diet are reached, appealing to its low risk of complications. In fact, a position paper by the International Study Group on Pancreatic Surgery (ISGPS) on nutritional support in patients undergoing pancreatic surgery was published in 2018 [110], and they recommended artificial nutritional support in patients who, although well nourished, would not tolerate an oral diet covering at least 50% of their caloric and protein requirements by 7th POD. Furthermore, the 2019 update of the ERAS guidelines [104] recommend considering postoperative artificial nutrition on an individualized basis depending on nutritional status, with a preference for the enteral route, with a moderate level of evidence and a strong grade of recommendation. This is supported by the study published in 2013 by Gerritsen et al. [111], a meta-analysis that demonstrated that oral diet combined with gastrojejunostomy reduced the length of hospital stay when compared with the use of jejunostomy, parenteral nutrition and nasojejunal tube. On the other hand, in the first group, the ability to perform a normal oral diet was also achieved earlier than in the other groups.

Regarding perioperative nutritional support in liver cancer, a meta-analysis evaluating the role of immunonutrition in patients undergoing hepatectomy was published in 2020 [112]. It included 11 clinical trials and showed a reduction in length of hospital stay (especially in patients with parenteral nutrition) and in infectious surgical complications, but not in mortality. Nevertheless, it was concluded that this type of formula should always be used in these patients in the context of the ERAS protocol.

### 5.2. Other Types of Cancer

Up to 60% of patients with head and neck cancer (HNC) present with significant malnutrition due to the oral feeding difficulties associated with this disease [113]. Between 20–50% of patients with high-risk surgical HNC will present with post-surgical complications such as wound infections, fistulas, anastomotic leaks and worse prognosis [114]. The most recent studies regarding perioperative nutrition in HNC have focused on the role that immunonutrition (especially arginine-rich formulas) might have in improving outcomes. In 2018, the Cochrane Library published a systematic review [115] which included 19 clinical trials (1099 patients) comparing the use of immunonutrients with the use of other formulas or not using them in patients with HNC. They found no significant differences in terms of wound infection or length of hospital stay, although they did find differences in relation to fistulas. In general, the quality of the studies was evaluated as low or very low, which is in agreement with previous meta-analyses by Stableforth et al. [116] and Vidal-Casariego et al. [117].

There is little evidence regarding perioperative nutrition in patients with urological tumors, despite the fact that up to 55% of patients undergoing radical cystectomy (RC) and/or urinary detour (DU) present with malnutrition [118], and that this has been associated with worse prognosis and increased mortality 90 days after surgery [119]. In 2013, Roth et al. [120] conducted a clinical trial with patients undergoing RC/DU comparing two groups: one had PN during the first five postoperative days and oral supplements and the other had only ONS. PN was associated with more complications in general and infectious complications in particular. In 2014, Bertrand et al. [121] compared historical controls with patients who were given immunonutrient formulas prior to surgery, finding a reduction in postoperative complications and infection. In 2016, Hamilton-Reeves et al. [122] confirmed these results with a clinical trial of 29 patients. In 2019, a Cochrane library review [123] evaluated the role of perioperative nutrition in patients with bladder cancer undergoing surgery with RC, including eight clinical trials. The main conclusion was that studies in this regard are currently of low or very low quality and that the evidence for benefit in these patients is very limited.

## 6. Perioperative Nutritional Support in Non-Oncological Pathology

### 6.1. Cardiac Surgery

Malnutrition in Congestive Heart Failure (CHF) is not an isolated or unidirectional phenomenon. It is estimated that 50% of patients with CHF have malnutrition, and between 19 and 39% cachexia [124,125]. CHF causes tissue hypoperfusion, creating and favoring a hypoxic environment and activation of the immune system with increased expression of pro-inflammatory cytokines (TNFα, IL-1, IL-6) that condition a situation of chronic inflammation that, in turn, as previously explained, causes an increase in catabolism and the appearance of sarcopenia and loss of muscle mass. TNFα induces myocyte apoptosis and proteolysis (along with IL-6), while decreasing hepatic albumin synthesis and inhibiting appetite, synergistically with IL-1. IL-6 also inhibits hepatic albumin synthesis. On the other hand, these patient develop a resistance to insulin, along with a decrease in insulin-like growth factor 1 (IGF-1) and testosterone and dehydroepiandrosterone (DHEAS), which ultimately translates into a net increase in catabolism and a reduction in anabolic phenomena, along with a reduction in metabolic expenditure [124,126,127,128,129,130,131,132,133]. Likewise, in CHF, the activation of various neurohormonal systems occurs. There is a chronic imbalance between the sympathetic and parasympathetic nervous systems, with a net increase in adrenaline and noradrenaline that induce catabolism. In addition, there is also activation of the renin-angiotensin-aldosterone system, with an increase in angiotensin II. Angiotensin II induces sarcopenia and loss of muscle mass by numerous mechanisms: Increased oxidative stress through nicotinamide adenine dinucleotide phosphate (NADPH) oxidase, decreased IGF-1 and increased pro-inflammatory cytokines (So it further worsens the pro-inflammatory situation), reduced appetite by direct effect on the central nervous system (CNS), alteration of metabolic expenditure by actions on adenosine monophosphate activated protein kinase (AMPK) and finally, but not least, decreased myocyte regeneration [126,131,132,134]. It should be remembered that the heart is mostly a muscular structure, so the tissue destruction generated by all exposed processes further worsens the situation of heart failure; in fact, sarcopenia itself is a cause of CHF, worsening cardiac mechanics, and is a factor of poor prognosis. Intestinal hypoperfusion and the presence of wall edema condition the appearance of malabsorptive phenomena that cause not only a lower absorption of nutrients, but a whole plethora of digestive symptoms that worsen even more if possible the already delicate clinical situation of these patients [124,125,126,127,128,130,133,135,136,137,138,139].

Cardiac cachexia is defined as an unintentional and edema-free weight loss greater than 5% in the last 12 months (or a body mass index < 20) (Major criterion), accompanied by at least three of the following findings (Minor criteria): decreased muscle strength, fatigue, anorexia, low lean mass index or laboratory alterations such as elevated inflammatory markers (C-reactive protein or IL-6), hemoglobin < 12 g/dL or serum albumin < 3.2 g/dL [125,128,129,130,135,140]. As we have previously anticipated, the appearance of this syndrome implies a worse prognosis in patients with CHF, with a mortality of 50% at 18 months, apart from a worse quality of life and functional capacity [125,133,134,141,142]. Thus, it is not surprising that patients who are going to undergo cardiac surgery are diagnosed with malnutrition in a high percentage (17–20% are at high risk of malnutrition and in some series, it reaches 46%, and even 50% in CHF) [19,38,143,144,145]. The presence of malnutrition is an independent risk factor for the appearance of postoperative complications and is associated with higher mortality and hospital stay [19,20,38,146].

The ERAS society’s guidance on cardiac surgery recommends preoperative nutritional screening in all patients, as well as the use of dietary counseling and nutritional support prior to surgery to reduce complications. In addition, it discourages the use of PN unless severe malnutrition is present [147]. Some authors propose the use of preoperative ONS, especially in patients with severe malnutrition, since these supplements appear to improve complication rates and hospital stay [38,144,145,146]. Rozentryt et al. [148] demonstrated in a randomized, double-blind pilot study that the use of ONS versus placebo improved weight (though mostly at the expense of fat mass) and quality of life, along with a significant reduction in circulating TNFα. In the Nutrition Effect on Unplanned Readmissions and Survival in Hospitalized Patients (NOURISH) study, which included malnourished patients with CHF, a significant reduction in mortality was found 90 days after hospital discharge (4.8% vs. 9.7%; relative risk 0.49, 95% confidence interval 0.27 to 0.90; *p* = 0.018) and decreased hospital readmissions specifically in patients with CHF (0.2 vs. 36.1%, *p* = 0.001) in those patients who were administered both in hospital and at home two specific ONS enriched in hydroxymethylbutyrate (which has a beneficial role on muscle and sarcopenia) [149,150,151]. In the Cologne Corona Surveillance (CoCoS) study, which included 351 patients who received preoperative nutritional support, the authors found substantial reductions in mortality and complications postoperatively, especially in women [152]. Therefore, the use of ONS preoperatively is most likely to be cost-effective. With regard to postoperative nutrition, the ERAS society guide recommends the early establishment of nutritional support, with control of postoperative nausea/vomiting [147].

### 6.2. Bariatric Surgery

Most centers recommend that immediately prior to surgery, patients follow a low-calorie or low-carbohydrate diet [153] to reduce liver size [154]. This has been associated with surgeons’ perceptions of a lower complexity of surgery as well as a lower rate of postoperative complications [155]. In addition, pre-surgery weight loss has been associated with subsequent weight loss, especially the higher the previous body mass index (BMI) [155]. Obesity is a risk pathology for nutritional deficiencies, especially anemia, folic acid, vitamin B12 and vitamin D deficiency. That is why preoperative diets should be supplemented with vitamins and minerals in order to adequate the nutritional status for surgery [153].

Following ERAS protocols, the use of liquids with carbohydrates 2–3 h before surgery and solids up to 6 h before anesthetic induction is also recommended and has been shown to be safe in these patients [155]. After the treatment, it is recommended to start a liquid diet with calcium, iron, vitamins and minerals supplementation after 4 h [155].

### 6.3. Spine and Hip Surgery

Low albumin, transferrin and lymphocyte levels have been associated with increased surgical wound infections, post-surgical complications, increased length of hospital stay, and 30-day readmission and mortality after spinal surgery. However, few studies have evaluated the role of nutrition in these patients [156]. In a clinical trial, the use of nutritional support with protein and carbohydrate powders before and after surgery was associated with a shorter hospital stay, a lower incidence of hydroelectrolyte disturbances and an increase in albumin levels on postoperative day 3 [157]. As in other surgeries, there are recommendations on perioperative nutrition made by the ERAS Society [156]. Liquids and solids are allowed up to 2 and 6 h, respectively, before surgery, with carbohydrate loading to attenuate insulin resistance and the catabolic state produced by surgery.

As for hip fracture surgery, it is more frequent in elderly patients, with a higher prevalence of malnutrition or high risk of malnutrition [158]. Mortality can reach up to 30% one year after fracture, and is higher in malnourished patients [158,159]. It has been shown that nutritional status tends to deteriorate further during hospitalization of these patients, especially if there is cognitive impairment [158]. Despite all this, perioperative nutritional support in hip fracture patients is still under debate. There are some studies that have shown benefits in terms of mortality [160], quality of life [161], or surgical infections [162]. However, other studies have not demonstrated clinical benefits of this support and have found that the tolerance of these patients to the nasogastric tube was low [163,164,165]. Therefore, more studies are needed in this regard.

## 7. Conclusions

Perioperative nutritional support, especially in malnourished patients or those at risk of malnutrition, reduces complications, hospital stay and mortality. The degree of evidence is variable, being more robust in head and neck oncological surgery, as well as digestive (esophageal, gastric and colonic) surgery, but in general, nutritional intervention can reduce postoperative complications, morbidity and mortality, as well as hospital stay. Every patient who is scheduled for major surgery should be evaluated from the nutritional point of view and those at risk should promptly receive proper support. Immunonutritional support should be considered in oncological pathology. Postoperatively, nutritional treatment should be started early in the most appropriate modality, and maintained at least until achieving functional restoration.

## Figures and Tables

**Table 1:** ESPEN recommendations on perioperative nutritional management.

Recommendation	Consensus Degree
1. Preoperative fasting from midnight is unnecessary in most patients. Patients undergoing surgery, who are considered to have no specific risk of aspiration, shall drink clear fluids until 2 h before anesthesia. Solids shall be allowed until 6 h before anesthesia (A).	97%
2. In order to reduce perioperative discomfort including anxiety oral preoperative carbohydrate treatment (instead of overnight fasting, the night before and 2 h before surgery) should be administered (B). To impact postoperative insulin resistance and length of stay (LOS), preoperative carbohydrates can be considered in patients undergoing major surgery (0).	100%
3. In most instances, oral nutritional intake shall be continued after surgery without interruption (A).	90%
4. It is recommended to adapt oral intake according to individual tolerance and to the type of surgery carried out with special caution to elderly patients (GPP).	100%
5. Oral intake, including clear liquids, shall be initiated within hours after surgery in most patients.	100%
6. It is recommended to assess the nutritional status before and after major surgery (GPP).	100%
7. Perioperative nutritional support therapy is indicated in patients with malnutrition and those at nutritional risk. Perioperative nutritional therapy should also be initiated if it is anticipated that the patient will be unable to eat for more than five days perioperatively. It is also indicated in patients expected to have low oral intake and who cannot maintain above 50% of the recommended intake for more than seven days. In these situations, it is recommended to initiate nutritional support therapy (preferably by the enteral route e oral nutritional supplements e tube feeding) without delay (GPP).	92%
8. If the energy and nutrient requirements cannot be met by oral and enteral intake alone (<50% of caloric requirement) for more than seven days, a combination of enteral and parenteral nutrition (PN) is recommended (GPP). PN shall be administered as soon as possible if nutrition therapy is indicated and there is a contraindication for enteral nutrition (EN), such as in intestinal obstruction (A).	100%
9. For the administration of PN, an all-in-one (three-chamber bag or pharmacy prepared) should be preferred instead of a multibottle system (B).	100%
10. Standard operative protocols for nutritional support are recommended to secure effective nutritional support therapy (GPP).	100%
11. Parenteral glutamine supplementation may be considered in patients who cannot be fed adequately enterally and, therefore, require exclusive PN (0).	76%
12. Postoperative PN including omega-3-fatty acids should be considered only in patients who cannot be adequately fed enterally and, therefore, require PN (B).	65%
13. Peri- or at least postoperative administration of specific formula enriched with (arginine, omega-3-fatty acids, ribonucleotides) should be given in malnourished patients undergoing major cancer surgery (B). There is currently no clear evidence for the sole use of these formulas enriched with immunonutrients vs. standard oral nutritional supplements (ONS) in the preoperative period (0).	89%
14. Patients with severe nutritional risk shall receive nutritional therapy prior to major surgery (A) even if operations including those for cancer have to be delayed (B). A period of seven to 14 days may be appropriate (0).	95%
15. Whenever feasible, the oral/enteral route shall be preferred (A).	100%
16. When patients do not meet their energy needs from normal food it is recommended to encourage these patients to take ONS during the preoperative period unrelated to their nutritional status (GPP).	86%
17. Preoperatively, ONS shall be given to all malnourished cancer and high-risk patients undergoing major abdominal surgery. A special group of high-risk patients are the elderly people with sarcopenia (A).	97%
18. Immune modulating ONS including (arginine, omega-3 fatty acids, and nucleotides) can be preferred (0) and administered for five to seven days preoperatively (GPP).	64%
19. Preoperative EN/ONS should preferably be administered prior to hospital admission to avoid unnecessary hospitalization and to lower the risk of nosocomial infections.	91%
20. Preoperative PN shall be administered only in patients with malnutrition or severe nutritional risk where energy requirement cannot be adequately met by EN (A) A period of 7–14 days is recommended (0).	100%
21. Early EN (within 24 h) shall be initiated in patients in whom early oral nutrition cannot be started, and in whom oral intake will be inadequate (<50%) for more than seven days (GPP). Patients undergoing major head and neck or gastrointestinal surgery for cancer (A); patients with severe trauma including brain injury (A); patients with obvious malnutrition at the time of surgery (A).	97%
22. In most patients, a standard whole protein formula is appropriate. For technical reasons with tube clotting and the risk of infection, the use of home-made diets for EN is not recommended in general (GPP).	94%
23. With special regard to malnourished patients, placement of a nasojejunal tube or NCJ should be considered for all candidates for EN undergoing major upper gastrointestinal and pancreatic surgery (B).	95%
24. EN shall be initiated within 24 h after surgery (A).	91%
25. It is recommended to start EN with a low flow rate (e.g., 10-max. 20 mL/h) and to increase the feeding rate carefully and individually due to limited intestinal tolerance. The time to reach the target intake can be very different and may take five to seven days (GPP).	85%
26. If long-term EN (>4 weeks) is necessary, e.g., in severe head injury, placement of a percutaneous tube (e.g., percutaneous endoscopic gastrostomy–PEG) is recommended (GPP).	94%
27. Regular reassessment of nutritional status during the stay in hospital and, if necessary, a continuation of nutritional support therapy including qualified dietary counseling after discharge, is advised for patients who have received nutritional support therapy perioperatively and still do not cover appropriately their energy requirements via the oral route (GPP).	97%
28. Malnutrition is a major factor influencing outcome after transplantation, so monitoring of the nutritional status is recommended. In malnutrition, additional ONS or even EN is advised (GPP).	100%
29. Regular assessment of nutritional status and qualified dietary counselling shall be required while monitoring patients on the waiting list before transplantation (GPP).	100%
30. Recommendations for the living donor and recipient are no different from those for patients undergoing major abdominal surgery (GPP).	97%
31. After heart, lung, liver, pancreas, and kidney transplantation, early intake of normal food or EN is recommended within 24 h (GPP).	100%
32. Even after transplantation of the small intestine, EN can be initiated early but should be increased very carefully within the first week (GPP).	93%
33. If necessary EN and PN should be combined. Long-term nutritional monitoring and qualified dietary counseling are recommended for all transplants (GPP).	100%
34. Early oral intake can be recommended after bariatric surgery (0).	100%
35. PN is not required in uncomplicated bariatric surgery (0).	100%
36. In case of a major complication with relaparotomy, the use of a nasojejunal tube/NCJ may be considered (0).	87%
37. Further recommendations are not different from those for patients undergoing major abdominal surgery (0).	94%

ESPEN, European Society for Parenteral and Enteral Nutrition; A/B/0/GPP, class grading; GPP, Good Practice Points; NCJ, needle catheter jejunostomy.

## Data Availability

Not applicable.
